# Mobility of β-Lactam Resistance Under Bacterial Co-infection and Ampicillin Treatment in a Mouse Model

**DOI:** 10.3389/fmicb.2020.01591

**Published:** 2020-07-07

**Authors:** Alexander Laskey, Marie Ottenbrite, John Devenish, Mingsong Kang, Mirjana Savic, Susan Nadin-Davis, John Chmara, Min Lin, James Robertson, Kyrylo Bessonov, Simone Gurnik, Kira Liu, John H. E. Nash, Andrew Scott, Edward Topp, Jiewen Guan

**Affiliations:** ^1^Ottawa Laboratory, Fallowfield, Canadian Food Inspection Agency, Ottawa, ON, Canada; ^2^National Microbiology Laboratory, Public Health Agency of Canada, Guelph, ON, Canada; ^3^London Research and Development Centre, Agriculture and Agri-Food Canada, London, ON, Canada

**Keywords:** antibiotic resistance, plasmid transfer, gut microbiota, intestinal inflammation, antibiotic treatment

## Abstract

Ingestion of food- or waterborne antibiotic-resistant bacteria may lead to the dissemination of antibiotic-resistance genes in the gut microbiota and the development of antibiotic-resistant bacterial infection, a significant threat to animal and public health. Food or water may be contaminated with multiple resistant bacteria, but animal models on gene transfer were mainly based on single-strain infections. In this study, we investigated the mobility of β-lactam resistance following infection with single- versus multi-strain of resistant bacteria under ampicillin treatment. We characterized three bacterial strains isolated from food-animal production systems, *Escherichia coli* O80:H26 and *Salmonella enterica* serovars Bredeney and Heidelberg. Each strain carries at least one conjugative plasmid that encodes a β-lactamase. We orally infected mice with each or all three bacterial strain(s) in the presence or absence of ampicillin treatment. We assessed plasmid transfer from the three donor bacteria to an introduced *E. coli* CV601gfp recipient in the mouse gut, and evaluated the impacts of the bacterial infection on gut microbiota and gut health. In the absence of ampicillin treatment, none of the donor or recipient bacteria established in the normal gut microbiota and plasmid transfer was not detected. In contrast, the ampicillin treatment disrupted the gut microbiota and enabled *S*. Bredeney and Heidelberg to colonize and transfer their plasmids to the *E. coli* CV601gfp recipient. *E. coli* O80:H26 on its own failed to colonize the mouse gut. However, during co-infection with the two *Salmonella* strains, *E. coli* O80:H26 colonized and transferred its plasmid to the *E. coli* CV601gfp recipient and a residential *E. coli* O2:H6 strain. The co-infection significantly increased plasmid transfer frequency, enhanced Proteobacteria expansion and resulted in inflammation in the mouse gut. Our findings suggest that single-strain infection models for evaluating *in vivo* gene transfer may underrepresent the consequences of multi-strain infections following the consumption of heavily contaminated food or water.

## Introduction

Antimicrobial resistance (AMR) is a seminally important public health issue threatening the efficacy of all medicines used to treat bacterial infections (Davies and Davies, 2010; World Health Organization, 2012; Laxminarayan et al., 2013). In response to this challenge, many countries have developed national AMR action plans that seek to mitigate AMR development (World Health Organization, 2012, 2015; Government of Canada, 2015; U.S. The White House, 2015). Since antibiotic-resistant bacteria are in humans, agriculture and the environment, the United Nations Interagency Coordination Group on Antimicrobial Resistance emphasizes that a One Health approach is essential to meet AMR challenge (McEwen and Collignon, 2018). Key actions planned include a reduction in antimicrobial use in human medicine and in agriculture, and improved water sanitation and hygiene (Pruden et al., 2013). These coordinated actions will reduce the pressure for resistance selection across the One Health continuum, and reduce transmission to humans via the environment and via food consumption (Graham et al., 2014; Berendonk et al., 2015; Holmes et al., 2016; Tiedje et al., 2019; Wang et al., 2019).

A healthy body of literature documents the abundance and characteristics of antibiotic-resistant bacteria in food production systems, the presence of antibiotic residues in agricultural land, and how these vary with agricultural practice (Lau et al., 2017; Lhermie et al., 2019). Also, much information is available on antibiotic-resistant bacteria in terrestrial and aquatic systems and how these vary with the management of animal or human waste streams (Smalla et al., 2016; Larsson et al., 2018). In order to evaluate the risk of food- or waterborne contamination with antibiotic-resistant bacteria, it is critical to understand untoward consequences from ingestion of such bacteria. The unwanted consequences that may lead to development of antibiotic-resistant bacterial infection include at least the following four scenarios (Ashbolt et al., 2013). Antibiotic-resistant bacteria infect the host following ingestion. The ingested antibiotic-resistant bacteria establish in the host microbiota and serve as a reservoir for gene recruitment into a pathogen. Antibiotic-resistance genes are transferred from the ingested antibiotic-resistant bacteria into the host microbiota, which then serves as a reservoir for gene recruitment into a pathogen. Antibiotic-resistance genes are transferred directly into a pathogen in the host.

However, the host gut microbiota may hinder the antibiotic-resistance transmission. The gut microbiota provides colonization resistance against pathogens or exogenously introduced bacteria through competition for niches and nutrients, contact-dependent killing, and production of antagonistic molecules (Deriu et al., 2013; Maltby et al., 2013; Buffie et al., 2014). It also mediates colonization resistance through keeping the host intestinal epithelium in a state for the generation of a rapid defense response to these bacteria (Bevins and Salzman, 2011; Sassone-Corsi and Raffatellu, 2015). On the other hand, the use of antibiotics may cause dysbiosis, reduce colonization resistance, and facilitate antibiotic-resistant bacterial infection and subsequent inflammation development (Kang and Martin, 2017).

In this study, we used β-lactam resistance as an example of AMR. Resistance to β-lactam compounds in Gram negative bacteria is primarily due to the production of β-lactamases that hydrolyze and thereby inactivate β-lactam antibiotics (Bush and Jacoby, 2010). Genes encoding β-lactamases (*bla* genes) such as OXA, CMY, TEM, SHV, and CTX-M are highly associated with mobile genetic elements, in particular conjugative plasmids (van Hoek et al., 2011; Cantón et al., 2012; Martin et al., 2012; Rozwandowicz et al., 2018). Evidence on humans and food-animals sharing the same *bla* genes, plasmids and strains suggests possible transmission through food contamination (Winokur et al., 2001; Leverstein-van Hall et al., 2011; Kluytmans et al., 2013; Mitchell et al., 2015). Consumption of water or crops that are exposed to human or animal waste streams may be another important transmission pathway (Walsh et al., 2011; Finley et al., 2013; Blau et al., 2018; Leonard et al., 2018). Food or water may be contaminated with a mixture of antibiotic-resistant bacteria, but animal studies on resistance transmission were mainly based on infection models using single-strain of resistant bacteria (Schjørring et al., 2008; Faure et al., 2010; Stecher et al., 2012; Gottig et al., 2015; Aviv et al., 2016). Little is known about resistant transmission following ingestion of multiple resistant bacterial strains. In the present study, we characterized three antibiotic-resistant bacterial strains: *Escherichia coli* O80:H26 and *Salmonella enterica* serovars Bredeney and Heidelberg. We orally infected mice with each or all three bacterial strain(s) in the presence or absence of ampicillin treatment. We then assessed the mobility of β-lactam resistance and the impacts of the bacterial infection on gut microbiota and gut health.

## Materials and Methods

### Bacterial Strains

*Escherichia coli* O80:H26 (EC-107), *Salmonella enterica* Bredeney (SA20114778WT), and *Salmonella enterica* Heidelberg (SL-312) are multi-antibiotic resistant bacteria isolated from chicken and turkey farms (Table 1 and Supplementary Table S1). Individual strains or a mixture of the three bacteria were used as donors in the mouse experiments described below. The *E. coli* CV601gfp (O16:H48) strain carrying a green fluorescent protein gene in its chromosome was used as a recipient (Heuer et al., 2002).

**TABLE 1:** Donor and recipient bacteria.

Bacteria^1^	Resistance profile^2^	Accession numbers^3^	Plasmid profile^4^	Predicted mobility	Resistance gene encoded on plasmid
*Escherichia coli* O80:H26	Amc Amp Azi Faz Fot Fox Pod	CP043217-	IncI2	conjugative	*bla*_*CMY–2*_
(EC-107)	Taz Tio Axo Cep Str Sul Tet Sxt	CP043221	IncY	mobilizable	*aph(6)-*Id, *bla*_*TEM–1B*_, *str*A
			IncFII	conjugative	*aad*A2, *aph(3)-*Ia, *aph(6)-*Id, *dfr*A12, *mph*(A), *str*A, *sul*1, *tet*(A)
			ColRNAI	mobilizable	ND
			Inc-^5^	mobilizable	ND
*Salmonella* Bredeney	Amp Faz Fot Pod Taz Tio Axo	CP043222-	IncN	conjugative	*bla*_*CTX–M–1*_
(SA20114778WT)	Cep Gen Str Sul	CP043224	IncH	conjugative	*aad*A2, *ant*(2)-Ia, *sul*1
			Inc-	non-mobilizable	ND
*Salmonella* Heidelberg (SL-312)	Amc Amp Faz Fot Fox Pod Taz Tio Cep Chl Str Sul Tet Sxt	CP043214-CP043216	IncA/C2 IncX1	conjugative conjugative	*aph(3)-*Ia, *aph(3)-*Ib, *aph(6)-*Id, *bla*_*TEM–1B*_, *bla*_*CMY–2*_, *dfr*A1, *flo*R, *sul*1, *sul*2, *tet*(A) ND
			Inc-	mobilizable	ND
*Escherichia coli* CV601gfp (O16:H48)	Gen Kan		ND^6^		

*^1^*Escherichia coli* O80:H26 (EC-107), *Salmonella* Bredeney (SA20114778WT) and *Salmonella* Heidelberg (SL-312) were donor bacteria and *E. coli* CV601gfp recipient. ^2^Amc = Amoxicillin-Clavulanic acid, Amp = ampicillin, Faz = cefazolin, Fot = cefotaxime, Fox = cefoxitin, Pod = cefpodoxime, Taz = ceftazidime, Tio = ceftiofur, Axo = ceftriaxone, Cep = cephamycin, Chl = chloramphenicol, Str = streptomycin, Sul = sulfamethizole, Tet = tetracycline, Sxt = trimethoprim-sulphamethoxazole, Gen = gentamicin, Kan = kanamycin. ^3^Each accession range includes chromosomal and plasmid components of a single isolate. ^4^Plasmid profile was determined by whole genome sequencing and the MOB-suite tool. ^5^Inc- = plasmid with no detectable Inc type. ^6^ND = Not detected.*

**FIGURE 1:**
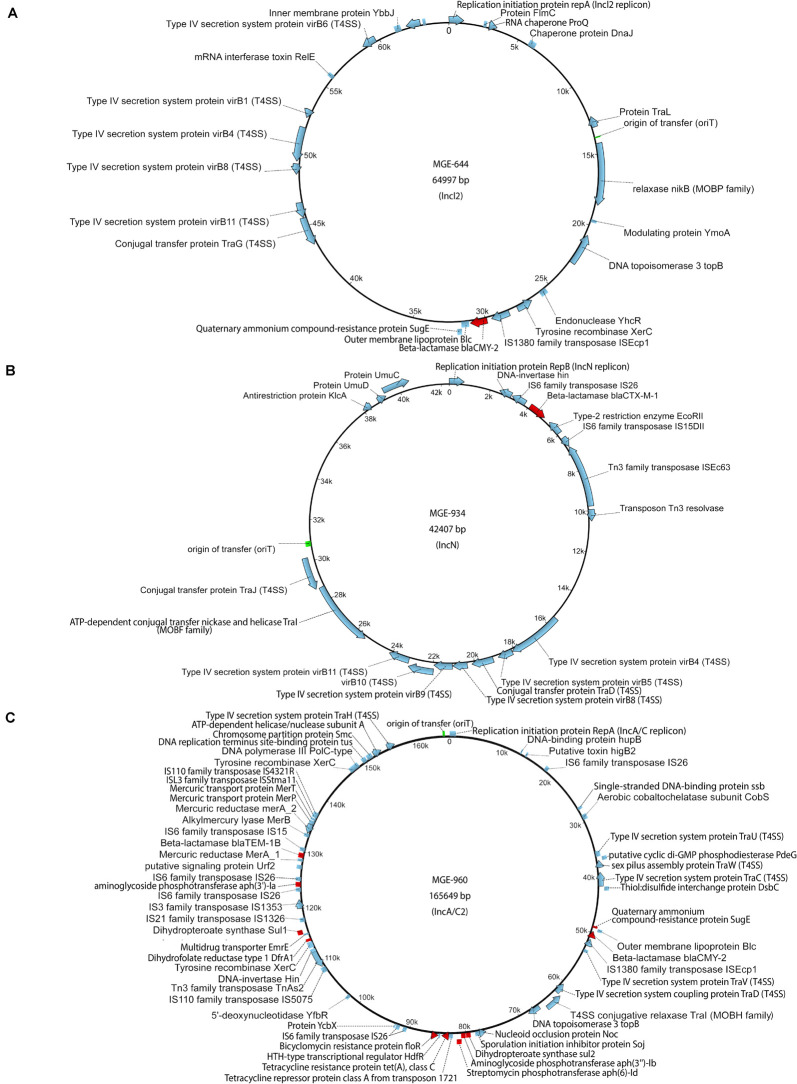
Maps of plasmids: **(A)** IncI2 plasmid carried by the *Escherichia coli* O80:H26 (EC-107) donor, **(B)** IncN plasmid carried by the *Salmonella* Bredeney (SA20114778WT) donor, **(C)** IncA/C2 plasmid carried by the *Salmonella* Heidelberg (SL-312) donor. AMR genes (red), origin of transfer (green) and other genes (blue).

Each donor strain carries three to five conjugative and mobilizable plasmids according to whole genome sequencing analysis and plasmid characterization with the MOB-suite tool (Table 1 and Supplementary Table S2). The resistance genes encoded on the plasmids may be responsible for the multi-antibiotic resistance of the bacteria. Three conjugative plasmids that encode β-lactamases were used as targets for evaluation of horizontal transfer. These plasmids were designated by their incompatibility type according to the replication initiation protein gene: an IncI2 plasmid (MGE-644) carried by *E. coli* O80:H26, an IncN plasmid (MGE-934) carried by *S*. Bredeney and an IncA/C2 plasmid (MGE-960) carried by *S.* Heidelberg (Figures 1A–C and Supplementary Table S2). All three plasmids possess an origin of transfer (oriT) and encode a relaxase or nickase, a type IV coupling protein and a set of type IV secretion system (T4SS) proteins. The IncI2 and IncN plasmids each carry one resistance gene, and the IncA/C2 plasmid carries ten resistance genes (Figures 1A–C). The β-lactamase genes are flanked by or in close proximity to transposase genes in all three plasmids.

### *In vitro* Conjugation

Donor strains were maintained on Chromocult agar (EMD Millipore, Toronto, ON) supplemented with 4 μg/mL cefotaxime (CHR-FOT), and the recipient strain was maintained on Chromocult agar supplemented with 50 μg/mL rifampicin and 50 μg/mL kanamycin (CHR-RK).

The donor strains were inoculated into Luria-Bertani (LB; Miller formulation, Difco, Thermo Fisher Scientific, Ottawa, ON, Canada) broth supplemented with cefotaxime (4 μg/mL final) and incubated with shaking at 30°C overnight. The recipient strain was inoculated into LB broth supplemented with rifampicin (50 μg/mL final) and kanamycin (50 μg/mL final) and incubated with shaking at 30°C overnight. The cultures were pelleted and washed three times by centrifugation at 3,000 × *g* for 10 min followed by resuspension in 1/10 × LB broth. After the final centrifugation, the cells were suspended to a final OD_600_ of 1.0 in 1/10 × LB broth. Conjugation was assessed as follows: to a 4.5 mL aliquot of 1/10 × LB broth, 500 μL of donor cell suspension was aseptically added, vortexed briefly, and 50 μL of recipient cells were aseptically added. The cells were vortexed briefly and incubated statically overnight at 30°C. Similar aliquots containing only donor strain or only recipient strain were also prepared and incubated under the same conditions.

After incubation, a 10-fold dilution series (10^–1^ to 10^–8^) of the cultures was prepared in sterile saline (0.85% NaCl, pH7.0). The donor:recipient crosses were plated in triplicate onto CHR-RK, CHR-FOT and Chromocult agar supplemented with 50 μg/mL kanamycin, 50 μg/mL rifampicin and 4 μg/mL cefotaxime (CHR-RKF) and incubated at 30°C for 48–72 h. Control cultures were plated similarly onto Chromocult agar, CHR-FOT, CHR-RK, and CHR-RKF and incubated under the same conditions. Recipient colonies were enumerated on CHR-RK (*E. coli* CV601gfp, indigo, green fluorescent) and donor colonies on CHR-FOT (*E. coli* O80:H26, indigo; *S*. Bredeney, turquoise; *S*. Heidelberg, cream), respectively. Transconjugant colonies were enumerated as indigo colonies producing green fluorescence under UV illumination (Blak-Ray B-100AP/R lamp, VWR International, Mississauga, ON, United States) on CHR-RKF. Conjugation frequencies are expressed as the ratio of enumerated transconjugants to enumerated donors.

### *In vivo* Conjugation

Experiments and procedures involving mice conformed to guidelines established by the Animal Care Committee at the Canadian Food Inspection Agency, Ottawa Laboratory (Fallowfield). Female C57BL/6 mice at the age of 28 days were purchased from Charles River Laboratories (Saint Constant, QC, Canada). Mice were mixed and acclimatized for 2 weeks prior to bacterial inoculation and/or antibiotic treatment, and then housed three or four per cage (Optimice^®^, Animal Care Systems, Centennial, CO, United States) with water and feed provided *ad libitum*. Independent experiments were carried out using various donor bacteria in the presence or absence of ampicillin treatment (Table 2) to investigate the transfer of plasmids carrying β-lactam resistance genes. Bacterial inocula (100 μL) containing ∼3.0 × 10^8^ colony forming units (CFU) of *E. coli* O80:H26, *S.* Heidelberg or Bredeney or the mixture of ∼1.0 × 10^8^ CFU of each of the three bacteria as donors was given to each mouse, followed by 100 μL of ∼3.0 × 10^8^ CFU of *E. coli* CV601gfp as recipients through oral gavage an hour later. Immediately following the bacterial inoculation, some mice (Table 2) were provided ampicillin in drinking water *ad libidum* (0.16 mg ampicillin/mL water, which is equivalent to a dosage of ∼30 mg/kg per day based on each mouse weighing 20 g and consuming an average of 5 mL water a day). Fecal pellets were collected from all mice on day 0 and 1, 2, and 5 day post infection (dpi). One set of pellets were immediately stored in dry-ice and then at −80°C for microbiome analysis and the other set kept in ice for bacterial culture. Within the collection day, pellets kept in ice were weighed and then homogenized in 1.0 ml phosphate-buffered saline (PBS, pH 7.2). The homogenates were 10-fold serially diluted in PBS and suspensions plated on Chromocult agar supplemented with antibiotics as described above to enumerate donors, recipients and putative transconjugants. Following 24 h incubation at 37°C, bacteria on plates were enumerated as described above. On 7 dpi, all mice were euthanized and tissues of small intestine, cecum and colon were collected and immediately stored in 10% neutral buffered formalin for histological examinations.

**TABLE 2:** Treatment groups in mouse experiments^1^.

Treatment	Donor	Recipient	Amp	*n*
Clt-Amp	No	No	Yes^3^	6
Ctl	No	No	No	6
EC-Amp	EC	Yes^2^	Yes	6
EC	EC	Yes	No	6
SB-Amp	SB	Yes	Yes	6
SB	SB	Yes	No	6
SH-Amp	SH	Yes	Yes	5
SH	SH	Yes	No	5
Mix-Amp	Mix	Yes	Yes	4
Mix	Mix	Yes	No	4

*^1^Ctl = control without bacterial inoculation, Amp = ampicillin, EC = *Escherichia coli* O80:H26 (EC-107), SB = *Salmonella* Bredeney (SA20114778WT), SH = *Salmonella* Heidelberg (SL-312), Mix = the mixture of EC, SB and SH, *n* = number of mice used in each treatment group. ^2^Yes = mice were inoculated with *Escherichia coli* CV601gfp as recipient 1 h after donor inoculation. ^3^Yes = mice were provided with ampicillin in drinking water (0.16 mg/mL) *ad libitum* immediately following bacterial inoculation.*

### Whole Genome Sequencing

Donor, recipient and putative transconjugant bacteria were characterized by whole genome sequencing. The sequence data were analyzed using the MOB-suite software tools (Robertson and Nash, 2018). Genomic DNA was isolated from the bacteria using the automated Qiagen EZ1 DNA tissue kit, according to manufacturer’s instructions, except 180 μL of G2 buffer was used with 10 μL of proteinase K and 10 μL of lysozyme (10 mg/mL; Sigma-Aldrich, Gillingham, United Kingdom). To characterize donor and recipient bacteria, PacBio and Illumina sequencing was used. PacBio sequencing was performed at the Génome Québec Innovation Centre (McGill University, Quebec, Canada) using single-molecule real-time (SMRT) cells in an RSII sequencer, which produced 120,000 to 150,000 reads per sample, with an average read length of 11,000 bp. Illumina sequencing on MiSeq version 3 (600-cycle kit, Illumina, MS-102-3003) using Nextera XT libraries (Illumina, FC-131-1031) was performed at the National Microbiology Laboratory (Guelph, ON, Canada) to a target of 60-fold coverage. All short and long read data was deposited under NCBI SRA study number SRP219110 under BioProject PRJNA560883, respectively. The final complete assemblies of genomes and plasmids were deposited under the accession numbers listed in Table 1.

The acquisition of *bla* genes by putative transconjugants was confirmed as follows. Representative colonies isolated from media plates for enumerating transconjugants (up to 3 colonies per plate) were subjected to whole genome sequencing using an Illumina MiSeq system and/or an Oxford Nanopore MinION sequencer (Oxford Nanopore, Cambridge, MA, United States) at the National Microbiology Laboratory (Guelph, ON, Canada). Illumina sequencing was as described above. Oxford Nanopore sequencing was performed according to the default manufacturer protocol for rapid barcoding. Samples were prepared using either SQK-RBK001 or SQK-RBK004 rapid barcoding kits and subsequently ran on a FLO-MIN106 R9.4 flow cell. Each multiplexed run produced between 4,719 and 111,488 reads per sample, with the mean read length ranging between 3,485 and 11,880 bp. Albacore v2.1.3 (Oxford Nanopore) was used to perform demultiplexing, base-calling and quality filtering of the raw reads.

Hybrid *de novo* assemblies of transconjugants were produced using the Unicycler pipeline v0.4.3 (Wick et al., 2017). Hybrid *de novo* assemblies of the recipient and donor strains were performed HGAP v3.1 PacBio assembly pipeline. All assemblies were manually reviewed to confirm completeness of the chromosome and any plasmids present. As part of the validation process, complete plasmid assemblies were mapped against raw reads using snippy (Seemann, 2015) pipeline to assess coverage and any potential coverage gaps. The assembled sequences were further analyzed using the MOB-suite (Robertson and Nash, 2018) and Prokka (Seemann, 2014) software tools. Plasmid maps were rendered using the UGENE software (Okonechnikov et al., 2012) and the plasmids were annotated using Prokka version 1.13.3.

### 16S rRNA Gene Amplicon Sequencing

DNA was extracted from each fecal pellet using the NucleoSpin^®^ Soil DNA extraction kit (Macherey-Nagel, Germany), per the manufacturer’s protocol. Concentrations of DNA samples were determined using a combination of the Qubit 2.0 Fluorometer (Thermo Fisher Scientific, Ottawa, ON, United States) with the Qubit dsDNA HS Assay Kit, as well as the Quanti-iT dsDNA Assay Kit (Thermo Fisher Scientific) with a BioTek FLx800 microplate fluorescence reader (Thermo Fisher Scientific). DNA libraries were prepared following Illumina’s 16S Metagenomic Sequencing Library Preparation protocol (Illumina). In brief, the V3-V4 region of the 16S ribosomal RNA gene was amplified through PCR (Klindworth et al., 2013) using KAPA HiFi HotStart Ready Mix (Roche, Cape Town, ZA, United States). PCR products were subsequently purified using Agencourt AMPure XP beads (Beckman Coulter, Mississauga, ON, United States) and indexed using primers from the Nextera XT Index Kit (Illumina, FC-131-2001). Indexed PCR products were purified and then subjected to quantitation and quality verification in the QIAxcel Advanced System with a QIAxcel DNA High Resolution Kit (Qiagen). Libraries were diluted accordingly, pooled, and denatured prior to loading into the MiSeq v3 Reagent Kit (Illumina, MS-102-3003) cartridge with approximately 15% PhiX control from the PhiX Control v3 Kit (Illumina, FC-110-3001). Libraries were sequenced using a MiSeq system (Illumina) at the Canadian Food Inspection Agency Ottawa Laboratory (Fallowfield). Raw read data was demultiplexed and then analyzed using Qiime2 (Bolyen et al., 2018) through a modified version of the Qiime2 pipeline created by Forrest Dusseault^1^. Denoising, filtering, and clustering of OTUs in Qiime2 was conducted using the DADA2 option. The biom file, tree file and metadata file generated from QIIME2 were combined into a phyloseq object using R package phyloseq^2^ for further analysis and visualization.

### Histology Analysis

Intestinal segments from small intestine, cecum and colon were prepared in Swiss rolls and fixed in 10% neutral buffered formalin for at least 24 h. Fixed tissues were embedded in paraffin, sectioned, and stained with hematoxylin and eosin (Fischer et al., 2008). Inflammation was quantitated by evaluating submucosal edema, PMN infiltration, goblet cell hyperplasia, and epithelial damage and given a total score from 0 – 4: 0, no disease; 1, minimal; 2, mild; 3, moderate; 4, marked as described by Erben et al. (2014).

### Statistical Analysis

Differences in the conjugation frequency or the relative fold change of Proteobacteria between treatment groups on the same sampling day, and differences in the relative abundance of each phylum or genus between sampling days within each treatment groups were tested using one-way ANOVA. Differences in the percentage of mice that developed inflammation in the cecum and colon between treatment groups were tested using the Fischer’s exact test. The correlation between the percentage of mice that developed inflammation and the relative fold change of the Proteobactia in the gut microbiota was tested using the Pearson correlation test. The treatment groups contain four to six mice (Table 2), and a mean value derived from technical triplicates from one fecal pellet of each mouse on each sampling date represents one data point. Data were analyzed using the GraphPad Prism 8.0 software (San Diego, CA, United States). A *P*-value < 0.05 was considered statistically significant.

## Results

### *In vitro* and *in vivo* Conjugation Potential

**FIGURE 2:**
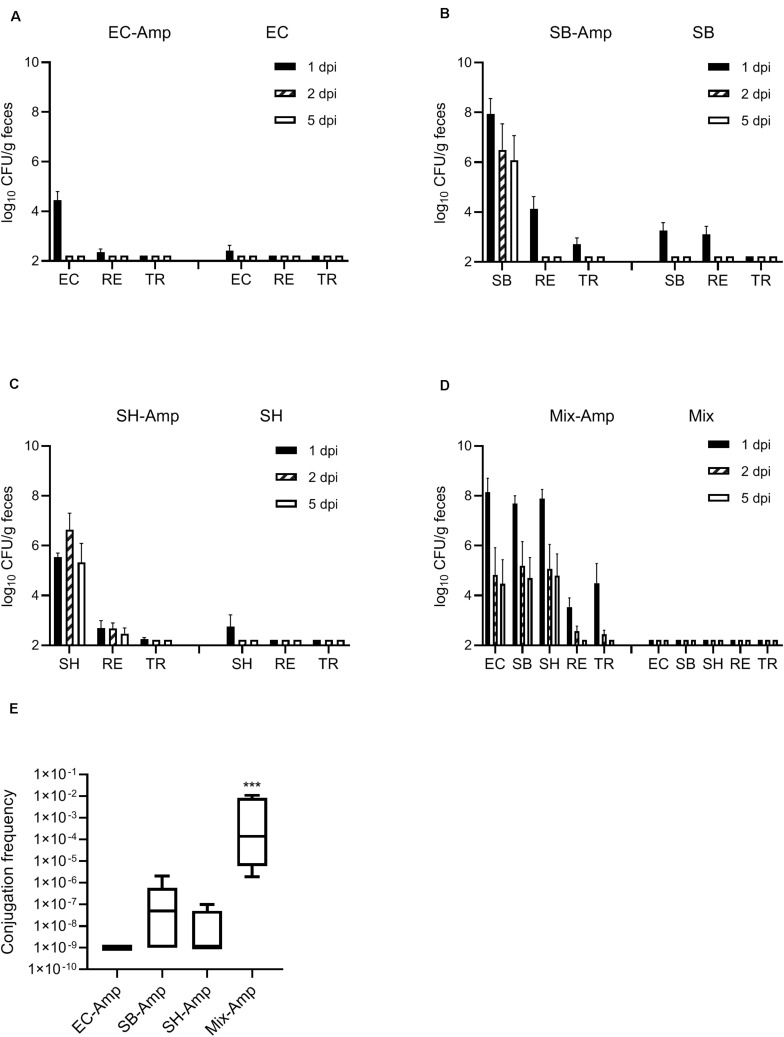
Enumeration of donor, recipient and transconjugant bacteria (mean + SE) in mouse fecal samples. Mice received the inoculation of donor bacteria: **(A)** EC = *Escherichia coli* O80:H26 (EC-107), number of mice (*n*) = 6. **(B)** SB = *Salmonella* Bredeney (SA20114778WT), *n* = 6. **(C)** SH = *Salmonella* Heidelberg (SL-312), *n* = 5. **(D)** Mix = the mixture of EC, SB and SH, *n* = 4 with (Amp) or without ampicillin treatment. RE = the *E. coli* CV601gfp recipient, TR = Transconjugants, dpi = day post infection. Conjugation frequency on 1 dpi **(E)** was expressed as the ratio of transconjugants to donors (sum of all three donors used in the Mix-Amp group). The conjugation frequency in the Mix-Amp group is significantly greater than that in other groups (*P* < 0.001) based on the one-way ANOVA test.

The *in vitro* conjugation frequency was 7.8 × 10^–4^, 5.3 × 10^–5^, and 3.0 × 10^–4^ between the *E. coli* O80:H26, *S*. Bredeney or *S.* Heidelberg donor and the *E. coli* CV601gfp recipient, respectively. This result indicated that the targeted β-lactam resistant plasmids were transferable, and therefore suitable for *in vivo* experimentation. In the presence of ampicillin treatment, shedding of the *S.* Bredeney and *S.* Heidelberg donors lasted for at least 5 days, with a maximum abundance in mouse feces at 1 day post infection (dpi) (Figures 2B,C). In comparison, shedding of the *E. coli* O80:H26 donor lasted for only 1 day, at 4.4 log_10_ CFU/g (Figure 2A). However, when co-introduced with the two *Salmonella* donor strains, shedding of the *E. coli* O80:H26 lasted for at least 5 days, with a maximum abundance of 8.1 log_10_ CFU/g at 1 dpi (Figure 2D). The recipient *E. coli* CV601gfp was shed for 1 or 2 days at a range of 2.6–4.1 log_10_ CFU/g. Fewer than 2.7 log_10_ CFU/g transconjugants were recovered, and only at 1 dpi from mice inoculated with a single donor strain (Figures 2A–C). In comparison, from mice inoculated with the mixture of all three donor strains, transconjugants were recovered at 1 and 2 dpi reaching 4.5 log_10_ CFU/g (Figure 2D), accompanied by some non-fluorescent colonies at 2 dpi. The conjugation frequency in mice with all three donor strains was significantly (*P* < 0.001) higher than that with each donor strain (Figure 2E), although each mouse received equal numbers of donor bacteria. In the absence of ampicillin treatment, transconjugants were not detected based on a detection limit of 2.2 log_10_ CFU/g. Shedding of the donors and recipient was less than 3.3 log_10_ CFU/g and lasted for only 1 day (Figures 2A–D).

### Confirmation of Horizontal Transfer of the Conjugative Plasmids

To confirm horizontal transfer of the β-lactam resistant plasmids, putative fluorescent and non-fluorescent transconjugants were subjected to whole genome sequencing analysis and plasmid characterization with the MOB-suite tool. Sequencing information on the representative transconjugants is available in BioProject PRJNA560883. According to the Mash distance analysis, plasmids in the transconjugants are identical to those in the corresponding donor bacteria, although there are a few mismatches likely due to sequencing errors (Supplementary Table S3). There were four different strains of transconjugants recovered from the mice that were inoculated with all three donor bacteria. Three strains were derived from the *E. coli* CV601gfp recipient and carried the IncI2, IncN and IncA/C2 plasmids, respectively. The other strain was a non-fluorescent *E. coli* O2:H6 carrying the IncI2 plasmid. Overall, the sequencing data confirmed the transfer of the conjugative plasmids from donor bacteria to the exogenously introduced *E. coli* CV601gfp and to an endogenously present *E. coli* O2:H6 recipient.

### Co-infection Promoted Inflammation in the Mouse Gut in the Presence of Ampicillin Treatment

**FIGURE 3:**
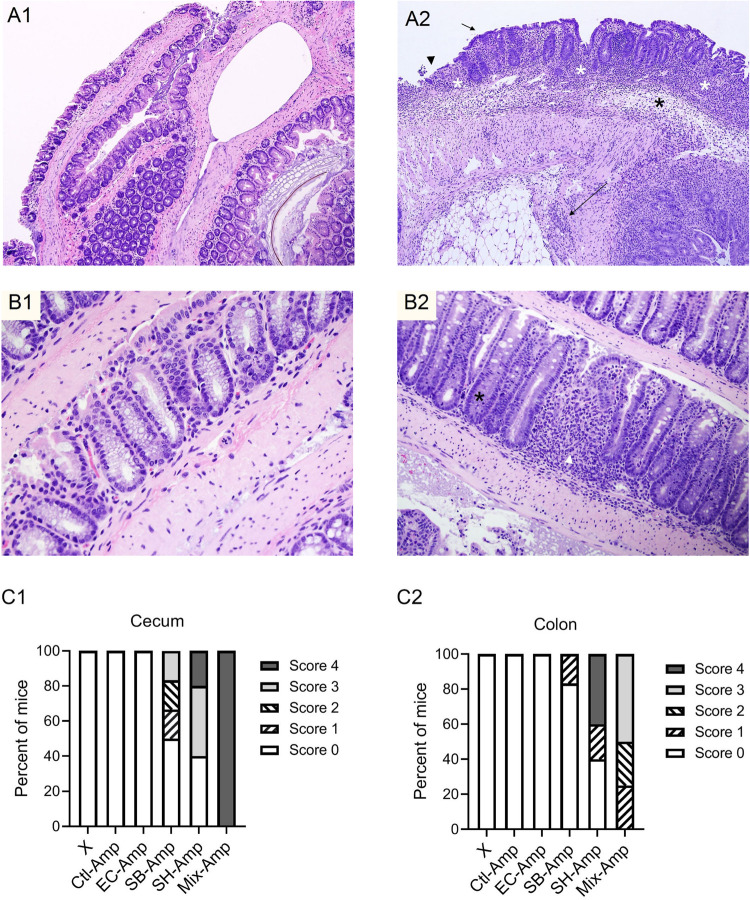
Representative images of hematoxylin and eosin (H&E)-stained cecum and colon sections. **(A1)** Normal cecum section with thin mucosa and extremely scant mononuclear cell infiltrate in lamina propria. **(A2)** Inflamed cecum section with markedly thickened mucosa and hyperplastic crypts, extensive crypt loss and proprial inflammatory cells (white asterisks), submucosal edema (black asterisk) and transmural inflammation (long arrow); surface epithelium is cuboidal and immature (short arrow) or eroded and ulcerated (arrowhead). **(B1)** Normal colon section with columnar surface epithelium and numerous mucous cells lining colonic crypts. **(B2)** Inflamed colon section with focal loss of crypts and proprial inflammation (white arrowhead), marked loss of mucous cells and crypt hyperplasia (black asterisk); and low columnar and more basophilic surface epithelium. Histologic inflammatory scores of the cecum **(C1)** and colon **(C2)** in each group of mice that received inoculation of donor bacteria and ampicillin treatment (Amp), Ctl = no bacteria, *n* = 6; EC = *Escherichia coli* O80:H26 (EC-107), number of mice (*n*) = 6; SB = *Salmonella* Bredeney (SA20114778WT), *n* = 6; SH = *Salmonella* Heidelberg (SL-312), *n* = 5; Mix = the mixture of EC, SB and SH, *n* = 4; or in all groups of mice (X) that received corresponding bacterial inoculation but no ampicillin treatment, *n* = 27. The percentage of mice that developed inflammation in the cecum or in the colon in the Mix-Amp group is significantly greater than that in the EC-Amp treatment group (*P* < 0.05) based on the Fischer’s exact test.

To determine if any of the treatments induced inflammation, intestine tissues were collected from each mouse on 7 dpi for histopathological analysis. Figures 3A1,A2,B1,B2 show the inflamed and normal cecum and colon tissues. In the presence of ampicillin treatment, inflammation was observed in the cecum of 0, 50, 60, and 100% of the mice infected by *E. coli* O80:H26, *S*. Bredeney, *S*. Heidelberg and the mixture of all three donor bacteria, respectively (Figure 3C1). Also, inflammation was observed in the colon of 0, 17, 60, and 100% of the mice from the corresponding treatment groups (Figure 3C2). The co-infection with all three donor bacteria resulted in significantly greater percentages of mice with inflammation in the cecum and colon compared to the infection with only *E. coli* O80:H26 (*P* < 0.05). No inflammation was found in mice receiving only ampicillin treatment but no bacterial inoculation, or only bacterial inoculation but no ampicillin treatment. In addition, no inflammation was found in the small intestine of all mice.

### Dynamics and Treatment Response of the Mouse Gut Microbiome

**FIGURE 4:**
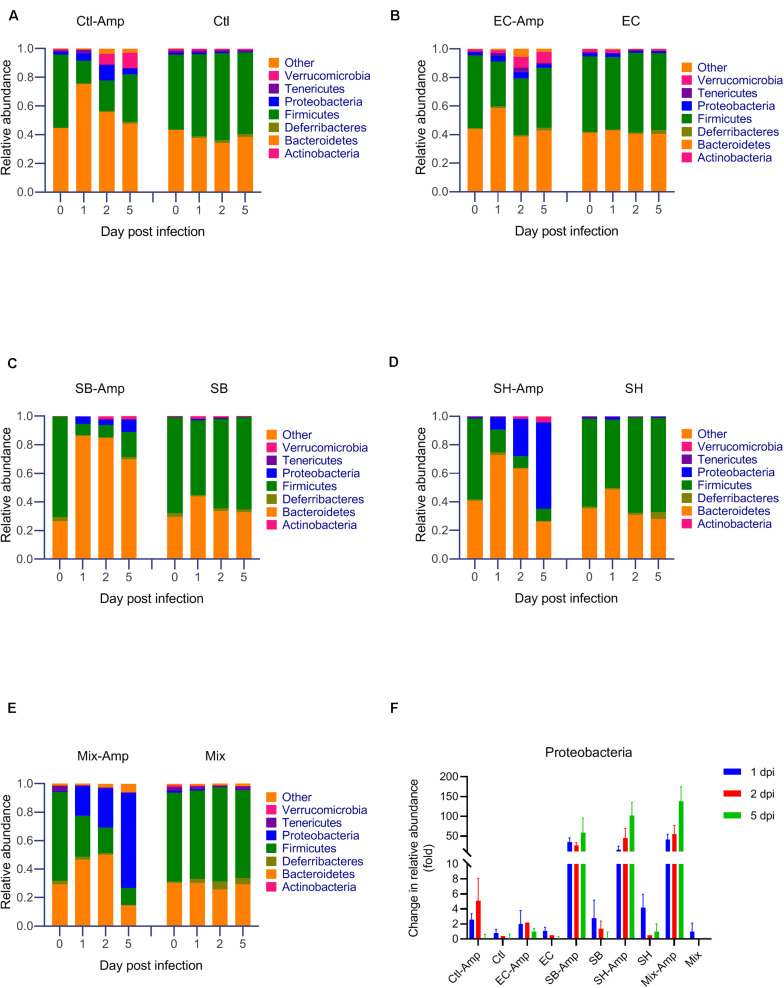
Microbial community composition analyzed by sequencing of the 16S rRNA gene from mouse fecal samples. Mice received the inoculation of donor bacteria: **(A)** Ctl = no bacteria, number of mice (*n*) = 6. **(B)** EC = *Escherichia coli* O80:H26 (EC-107), *n* = 6. **(C)** SB = *Salmonella* Bredeney (SA20114778WT), *n* = 6. **(D)** SH = *Salmonella* Heidelberg (SL-312), *n* = 5. **(E)** Mix = the mixture of EC, SB and SH, *n* = 4 with (Amp) or without ampicillin treatment. Data on the relative abundance of Proteobacteria from panels **(A–E)** were used to determine the relative changes of Proteobacteria responding to various treatments and expressed as the ratio of the relative abundance on 1, 2 or 5 day post infection (dpi) to that on day 0 within each treatment group (panel **F**). The relative abundance of Firmicutes, Bacteroidetes and Proteobacteria on 1 dpi is significantly different from that on day 0 within each ampicillin treatment group (*P* < 0.05). The relative fold change of Proteobacteria in the SB-Amp, SH-Amp or Mix-Amp treatment group is significantly greater than that in the Ctl-Amp or EC-Amp group on 1, 2 or 5 dpi (*P* < 0.05) based on the one-way ANOVA test.

**FIGURE 5:**
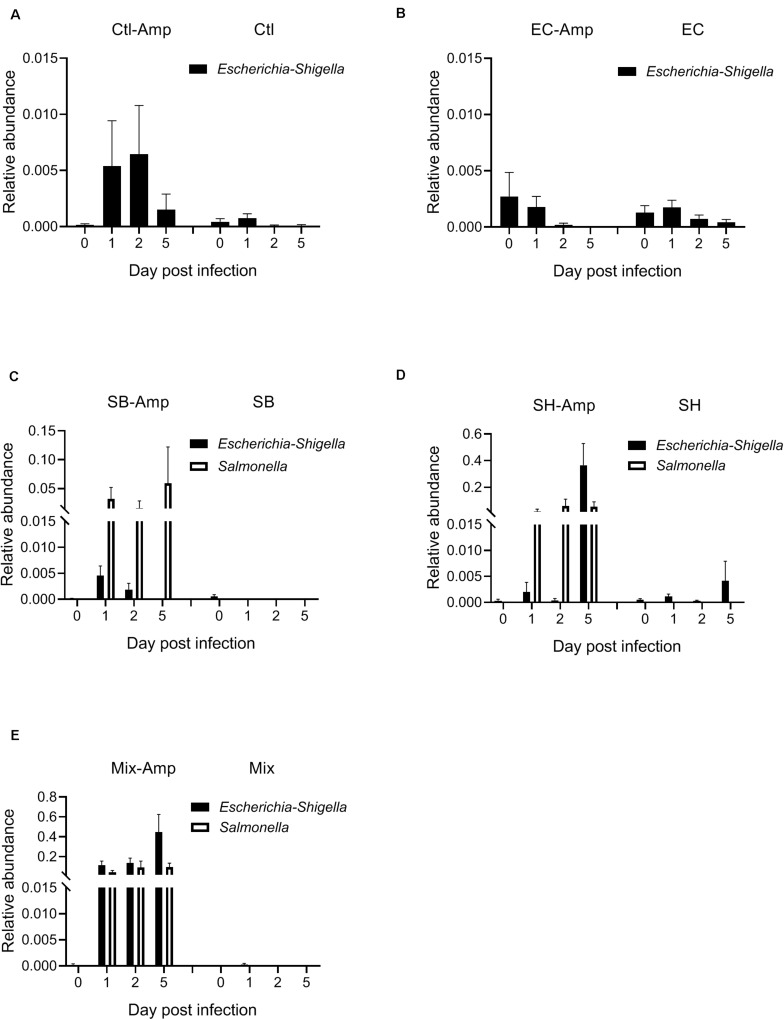
Relative abundance of the genera of *Escherichia*-*Shigella* and *Salmonella* in the mouse gut microbiome. Mice received the inoculation of donor bacteria: **(A)** Ctl = no bacteria, number of mice (*n*) = 6. **(B)** EC = *Escherichia coli* O80:H26 (EC-107), *n* = 6. **(C)** SB = *Salmonella* Bredeney (SA20114778WT), *n* = 6. **(D)** SH = *Salmonella* Heidelberg (SL-312), *n* = 5. **(E)** Mix = the mixture of EC, SB and SH, *n* = 4 with (Amp) or without ampicillin treatment. The relative abundance of *Escherichia*-*Shigella* on 1, 2 or 5 dpi is significantly greater than that on day 0 in the Mix-Amp treatment group (*P* < 0.05) based on the one-way ANOVA test.

Fecal samples from all mice were subjected to the 16S rRNA gene amplicon sequencing analysis in order to determine if treatments promoted dysbiosis. In the absence of ampicillin treatment, the composition of gut microbiome in mice with or without bacterial inoculation was relatively stable. The microbial community was dominated by Firmicutes and Bacteroidetes (Figures 4A–E). As expected, ampicillin treatment disrupted the gut microbiome, with a significant (*P* < 0.05) decrease in the relative abundance of Firmicutes and a significant (*P* < 0.05) increase in that of Bacteroidetes and Proteobacteria occurring at 1 dpi (Figure 4A). The introduction of various donor bacteria had differential impacts on the alteration of the microbial composition and diversity induced by the ampicillin treatment (Figures 4B–E and Supplementary Figures S1, S2). Colonization of *E. coli* O80:H26 slightly alleviated the dysbiosis, while that of *S*. Bredeney, *S*. Heidelberg and the mixture of all three donor bacteria aggravated the dysbiosis. To illustrate the differential impacts, the alteration of Proteobacteria relative abundance at 1, 2, and 5 dpi compared to that at day 0 were expressed as fold changes within each mouse group (Figure 4F). The fold changes in the mice with *E. coli* O80:H26 inoculation and ampicillin treatment were comparable to or slightly lower than those in the mice without bacterial inoculation or ampicillin treatment. In contrast, the fold changes in the mice with *S*. Bredeney, *S*. Heidelberg or the mixture of all three bacterial inoculation plus ampicillin treatment were significantly (*P* < 0.05) higher compared to those of the mice with only ampicillin treatment but no bacterial inoculation (Figure 4F). The mean fold changes in the relative abundance of Proteobacteria at 5 dpi were 1, 58, 102, and 138 for the mice inoculated with *E. coli* O80:H26, *S*. Bredeney, *S*. Heidelberg and the mixture of all three bacteria plus ampicillin treatment, respectively. These changes were positively correlated with the percentage of mice that developed intestinal inflammation in the corresponding treatment groups (*R*^2^ > 0.93, *P* < 0.01, Supplementary Figure S3). Since the donor and the recipient bacteria belong to the *Escherichia-Shigella* and *Salmonella* genera, the relative abundance of these two genera was analyzed. In the mice with all three bacterial inoculation and ampicillin treatment, the relative abundance of *Escherichia-Shigella* increased significantly (*P* < 0.05) from 0.0002 at day 0 to 0.1155, 0.1379, and 0.4464 at 1, 2, and 5 dpi, respectively (Figure 5E). However, in the mice with only *E. coli* O80:H26 inoculation and ampicillin treatment, the relative abundance of *Escherichia-Shigella* remained below 0.0027 over the course of the study (Figure 5B). Comparing data from these two treatment groups, there seemed to be an association between the relative abundance of *Escherichia-Shigella* and the colonization of *E. coli* O80:H26 and also the transfer of the IncI2 plasmid (Figures 2A,D, 5B,E). In comparison, the relative abundance of the *Salmonella* genus was greater than 0.0150 at 1, 2, and 5 dpi in the mice with *Salmonella* inoculation and ampicillin treatment (Figures 5C–E). In the absence of ampicillin treatment, the abundance of these two genera remained relatively stable and below 0.0040 in all mice (Figures 5A–E).

## Discussion

Building on previous animal studies on horizontal transfer of β-lactam resistance genes (Schjørring et al., 2008; Faure et al., 2010; Gottig et al., 2015), this study evaluated the impacts on resistance mobility from infection with single- versus multi-strain of resistant bacteria. We infected mice with each or all of the three β-lactam resistant *E. coli* and *Salmonella* bacteria in the presence of ampicillin treatment. Co-infection with all three bacteria significantly enhanced plasmid transfer in the mouse gut relative to single-strain infections. In addition, the co-infection exacerbated the ampicillin induced dysbiosis and promoted inflammation in the mouse cecum and colon.

The normal gut microbiota contributes to colonization resistance against exogenously introduced bacteria. It competes with exogenous bacteria for space, nutrients and host receptors, and promotes host immunity to these bacteria (Bevins and Salzman, 2011; Deriu et al., 2013; Maltby et al., 2013; Buffie et al., 2014; Sassone-Corsi and Raffatellu, 2015). Thus, in the absence of ampicillin treatment, the three introduced β-lactam resistant *E. coli* and *Salmonella* bacteria transiently passed through the gut and disappeared from the feces shortly after their introduction (≤1 day). To overcome colonization resistance this study applied antibiotic treatment, similar to other animal studies on gene transfer (Faure et al., 2010; Stecher et al., 2012; Aviv et al., 2016). We found differential colonization of *E. coli* O80:H26 on its own versus with *Salmonella*. Our data suggest that introduction of a mixture of resistant bacteria could have synergetic effects that enhance bacterial colonization in the gut microbiota.

In this study, co-infection with all three donor bacteria under ampicillin treatment significantly increased the resistance transfer frequency. The IncI2 plasmid was transferred from *E. coli* O80:H26 to both the introduced *E. coli* CV601-GFP and residential *E. coli*. Possibly, the co-infection enabled the high density colonization of *E. coli* O80:H26, and subsequently favored the transfer of the IncI2 plasmid. The high relative abundance of the *Escherichia*-*Shigella* genus (0.1155 ∼ 0.4464) under these circumstances might promote more frequent cell-cell contact between the donor and recipient *E. coli* for plasmid transfer in the gut microbiota. Likewise, the transfer of the IncN and IncA/C2 plasmids occurred in the presence of high density colonization of the *Salmonella* donor bacteria when the relative abundance of the Salmonella genus was high (≥0.015) in the gut microbiome. Conjugation in Gram negative bacteria requires cell-cell contact between competent donors and recipients, a small fraction of cells among the populations of donors and recipients (Koraimann and Wagner, 2014). Thus, high density colonization of donor and recipient bacteria would promote conjugative gene transfer in the gut microbiota. Overall, gene transfer may be more dynamic in instances where there are multi-strain infections and dysbiosis. On this basis we suggest that experiments with single-strain infections may underrepresent the consequences of consuming water or food that is contaminated with a range of bacteria carrying mobile plasmids.

Finally, this study evaluated the health impacts from the bacterial infection in the mouse gut. Gut health heavily relies on the homeostasis between the host immune system and the gut microbiota. Dysbiosis and bacterial infection may undermine the gut homeostasis and lead to the development of inflammation (Kang and Martin, 2017). Shi et al. (2018) showed that a 14-day ampicillin treatment induced dysbiosis in the microbiota which was accompanied by inflammatory reactions in the mouse gut. In comparison, in this study the 7-day ampicillin treatment itself caused mild dysbiosis but did not induce inflammation detectable by histological analysis. However, in the presence of ampicillin treatment, the co-infection significantly increased the Proteobacteria relative abundance and the percentage of mice with intestinal inflammation, relative to the single-strain infection with *E. coli* O80:H26. Dysbiosis with increased Proteobacteria and decreased Firmicutes could lead to reduced production of short chain fatty acids and weakened intestinal integrity, and thus would initiate intestinal inflammation (Cani et al., 2008; Shi et al., 2018). Overall, our findings suggest that co-infection with resistant bacteria might promote intestinal inflammation under antibiotic treatments.

## Conclusion

This study is the first to evaluate impacts from infection with single- versus multi-strain of resistant bacteria on resistance mobility under antibiotic selection pressure. Using a mouse model in the presence of ampicillin treatment, our study demonstrated that the co-infection with all three β-lactam resistant bacteria, *E. coli* O80:H26, *S*. Bredeney and *S*. Heidelberg significantly increased plasmid transfer frequency and enabled plasmid transfer into both introduced and residential *E. coli* strains. Furthermore, the co-infection induced dysbiosis in the gut microbiota and promoted intestinal inflammation. Our findings suggest that single-strain infection models for evaluating *in vivo* gene transfer may underrepresent the consequences of multi-strain infections following the consumption of food or water contaminated with a mixture of antibiotic-resistant bacteria.

## Data Availability Statement

The datasets generated for this study can be found in the NCBI BioProject PRJNA560883.

## Ethics Statement

The animal study was reviewed and approved by the Animal Care Committee at the Canadian Food Inspection Agency, Ottawa Laboratory (Fallowfield), Ottawa, ON, Canada.

## Author Contributions

JG, ET, and JN designed the experiments. JG wrote the manuscript, all co-authors edited and contributed to the revisions. AS and ET carried out *in vitro* plasmid transfer tests. AL, MO, JD, MK, ML, and JG carried out the animal experiments and bacterial enumeration and analyzed the bacterial culture data. MS performed the histology analysis. JC, SN-D, MK, and JG conducted the 16S rRNA gene amplicon sequencing analysis. SG and KL assisted with the whole genome sequencing. KB, JR, and JN analyzed the whole genome sequencing data. All authors contributed to the article and approved the submitted version.

## Conflict of Interest

The authors declare that the research was conducted in the absence of any commercial or financial relationships that could be construed as a potential conflict of interest.

## Footnotes

1 https://github.com/forestdussault/AmpliconPipeline

2 https://github.com/joey711/phyloseq
