# Supplementary material for: Mobility of β-Lactam Resistance Under Bacterial Co-infection and Ampicillin Treatment in a Mouse Model
Source: Front Microbiol. 2020 Jul 7;11:1591. doi: 10.3389/fmicb.2020.01591 (PMC7358583; doi:10.3389/fmicb.2020.01591)
Supplement: Supplementary file 1 [file Data_Sheet_1.pdf]

Supplementary table 1 (S1). Donor bacterial minimal inhibitory concentration (MIC)

Antibiotic	MIC (µg/mL) in[1]		
	Escherichia coli O80:H26 (EC-107)	*Salmonella* Bredeney (SA20114778WT)	*Salmonella* Heidelberg (SL-312)
Amoxicillin-Clavulanic acid	**32**	16	>**32**
Ampicillin	>**32**	>**32**	>**32**
Azithromycin	>**16**	4	4
Cefazolin	>**16**	>**16**	>**16**
Cefotaxime	**16**	>**64**	**16**
Cefoxitin	>**64**	4	**64**
Cefpodoxime	>**32**	>**32**	>**32**
Ceftazidime	**32**	**8**	**16**
Ceftiofur	>**8**	>**8**	>**8**
Ceftriaxone	**32**	>**128**	16
Cephamycin	>**16**	>**16**	>**16**
Chloramphenicol	4	4	>**32**
Gentamicin	0.5	>**16**	0.5
Streptomycin[2]	>**64**	**64**	>**64**
Sulfamethoxazole	>**256**	>**256**	>**256**
Tetracycline	>**32**	<4	>**32**
Trimethoprim-Sulfamethoxazole	>**4**	<0.12	>**4**

[1]Values in boldface interpreted as resistant per CLSI document M100-S26 (Clinical and Laboratory Standards Institute. 2016. Performance standards for antimicrobial susceptibility testing—26th ed. CLSI document M100-S26. Clinical and Laboratory Standards Institute, Wayne, PA.)

[2]No CLSI guidelines for streptomycin; breakpoint from the Canadian Integrated Program for Antimicrobial Resistance Surveillance, based on the MIC distribution of population data, was used.

Supplementary table 2 (S2). Characteristics of plasmids in donor bacteria[1]

Isolate	Plasmid ID	Length (bp)	GC (%)	Rep type	Rel type	MPF type	oriT type	Predicted Mobility	Mash nearest neighbor	Mash nearest neighbor distance	Resistance gene
ET6	MGE-1009	102028	49.2	IncY	MOB$_{P}$	-	-	Mobilizable	SRR3115211	0.0329	*aph*(6)-Id, *bla*$_{TEM-1B}$, *str*A
ET6	MGE-528	94175	53.9	IncFII	MOB$_{F}$	MPF$_{F}$	MOB$_{F}$	Conjugative	SRR5207260	0.0344	*aad*A2, *aph*(3)-Ia, *aph*(6)-Id, *dfr*A12, *mph*(A), *str*A, *sul*1, *tet*(A)
ET6	MGE-644	64997	42.4	IncI2	MOB$_{P}$	MPF$_{T}$	-	Conjugative	JN983044	0.0002	*bla*$_{CMY-2}$
ET6	MGE-17	2285	47.7	-	-	-	MOB$_{P}$	Mobilizable	SRR3098660	0.0391	-
ET6	MGE-415	1552	51.7	ColRNAI	-	-	-	Mobilizable	SRR5215867	0.0026	-
ET1	MGE-4152	39740	34.6	-	-	-	-	Non-mobilizable	CP021954	0.0375	-
ET1	MGE-430	257405	45.5	IncH	MOB$_{H}$	MPF$_{F}$	MOB$_{H}$	Conjugative	KM396298	0.0101	*aad*A2, *ant*(2)-Ia, *sul*1
ET1	MGE-934	62029	50.5	IncN	MOB$_{F}$	MPF$_{T}$	MOB$_{F}$	Conjugative	NC_019098	0.0047	*bla*$_{CTX-M-1}$
ET8	MGE-53	37697	41.2	IncX1	MOB$_{P}$	MPF$_{T}$	-	Conjugative	CP012922	2.38E-05	-
ET8	MGE-159	3046	44.9	-	-	-	MOB$_{P}$	Mobilizable	CP016527	0.0005	-
ET8	MGE-960	165649	52.6	IncA/C2	MOB$_{H}$	MPF$_{F}$	MOB$_{P}$	Conjugative	CP014658	0.0012	*aph*(3)-Ia, *aph*(3)-Ib, *aph*(6)-Id, *bla*$_{CMY-2}$, *bla*$_{TEM-1B}$, *dfr*A1, *flo*R, *sul*1, *sul*2, *tet*(A)

[1]Sequencing data are available in BioProject PRJNA560883. ET1 = *Salmonella* Bredeney (SA20114778WT), ET6 = *Escherichia coli* O80:H26 (EC-107), ET8 = *Salmonella* Heidelberg (SL-312), Rep = replication initiation protein, Rel = relaxase, MPF = mating pair formation, oriT = origin of transfer, - = non-detectable

Supplementary table 3 (S3) Genomic pairwise distance between plasmids isolated from donors and transconjugants

Plasmid	Donor	Transconjugant	Accession numbers[1]	MASH distance[2]	Identity (%)[3]
IncI2	*E. coli* O80:26	*E. coli* CV601gfp	CP043211-CP043212	0 (1000/1000)	100 (64997/64997)
		E. coli CV601gfp	CP043209-CP043210	0 (1000/1000)	100 (64997/64997)
		E. coli CV601gfp	CP043207-CP043208	0 (1000/1000)	100 (64997/64997)
		E. coli CV601gfp	CP043205-CP043206	0 (1000/1000)	100 (64997/64997)
		E. coli O2:H6	CP043203-CP043204	1.91626e-04 (992/1000)	99.9 (64979/64997)
		E. coli CV601gfp	CP043201-CP043202	1.43503e-04 (994/1000)	99.1 (64415/64997)
		E. coli CV601gfp	CP043211-CP043212	0 (1000/1000)	100 (64997/64997)
		E. coli CV601gfp	CP043197-CP043198	0 (1000/1000)	99.9 (64996/64997)
		E. coli CV601gfp	CP043195-CP043196	0 (1000/1000)	99.9 (64996/64997)
		E. coli CV601gfp	CP043193-CP043194	0 (1000/1000)	99.9 (64996/64997)
IncA/C2	*S.* Heidelberg	*E. coli* CV601gfp	CP043191-CP043192	0 (1000/1000)	99.9 (165646/165649)
		E. coli CV601gfp	CP043189-CP043190	0 (1000/1000)	99.9 (165646/165649)
IncN	*S.* Bredeney	*E. coli* CV601gfp	CP043187-CP043188	4.76906e-05 (998/1000)	98.1 (41601/42407)
		E. coli CV601gfp	CP043185-CP043186	7.15897e-05 (997/1000)	98.1 (41601/42407)
		E. coli CV601gfp	CP043183-CP043184	5.32603e-04 (978/1000)	98.1 (41601/42407)

1. Transconjugant sequence accession numbers, each range includes chromosomal and plasmid component(s) of a single isolate
2. Fast genome distance estimation based on MinHash using MASH v.2.1 (https://github.com/marbl/Mash) with default settings. In parenthesis the ratio of number of shared hashes between transconjugant and donor plasmids
3. Ratio between the number of identical nucleotide positions in plasmid in transconjuant against reference plasmid total length

Supplementary figure legend

Figure S1. Microbial richness and evenness as assessed by the Chao1, Shannon and Simpson indexes on each sampling date from various treatment groups: Ctl = control or no bacterial inoculation, EC = *E. coli* O80:H26 (EC-107), SB =*S*. Bredeney (SA20114778WT), SH = *S*. Heidelberg (SL-312), Mix = EC, SB and SH with (Amp) or without ampicillin treatment.

Figure S2. Principal coordinate analysis (PCoA) of weighted Bray-Curtis dissimilarity of mouse gut microbiome derived from individual mouse on each sampling date from various treatment groups: (A) no bacterial inoculation but with ampicillin treatment (Ctl-Amp), (B) *E. coli* O80:H26 (EC-107) and ampicillin treatment (EC-Amp), (C) *S*. Bredeney (SA20114778WT) and ampicillin treatment (SB-Amp), (D) *S*. Heidelberg (SL-312) and ampicillin treatment (SH-Amp), (E) EC, SB, SH and ampicillin treatment (Mix-Amp). Data derived from mice without ampicillin treatment were not shown as all data points cluster closely within each treatment group.

Figure S3. Correlation analysis between the percentage of mice in various treatment groups that developed inflammation in the cecum or colon and the mean relative fold change of the Proteobacteria relative abundance in the gut microbiome at 5 day post infection.
