# Supplementary figures and images for: Mobility of β-Lactam Resistance Under Bacterial Co-infection and Ampicillin Treatment in a Mouse Model
Source: Front Microbiol. 2020 Jul 7;11:1591. doi: 10.3389/fmicb.2020.01591 (PMC7358583; doi:10.3389/fmicb.2020.01591)

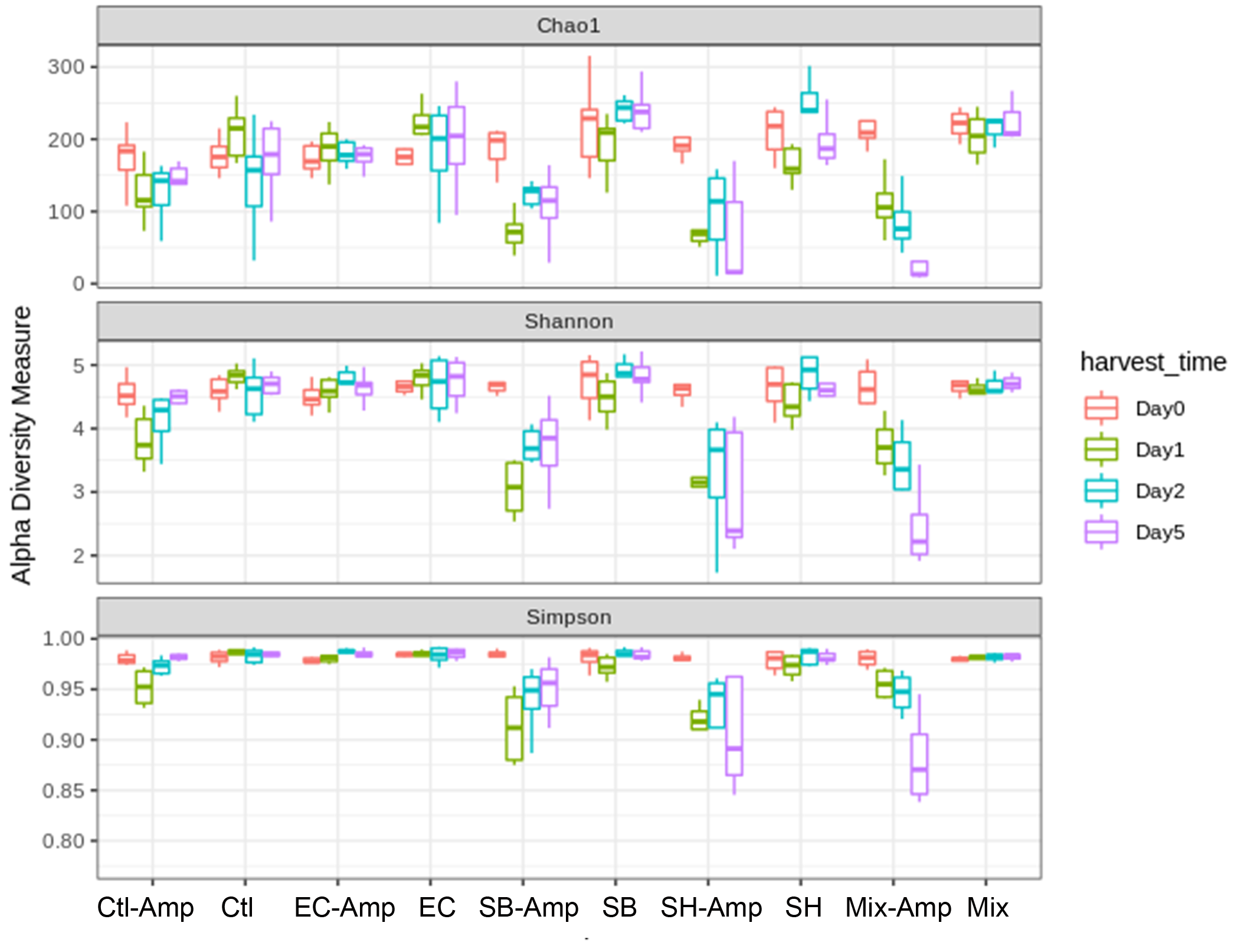

Supplement: Supplementary file 2 [file Image_1.TIF]

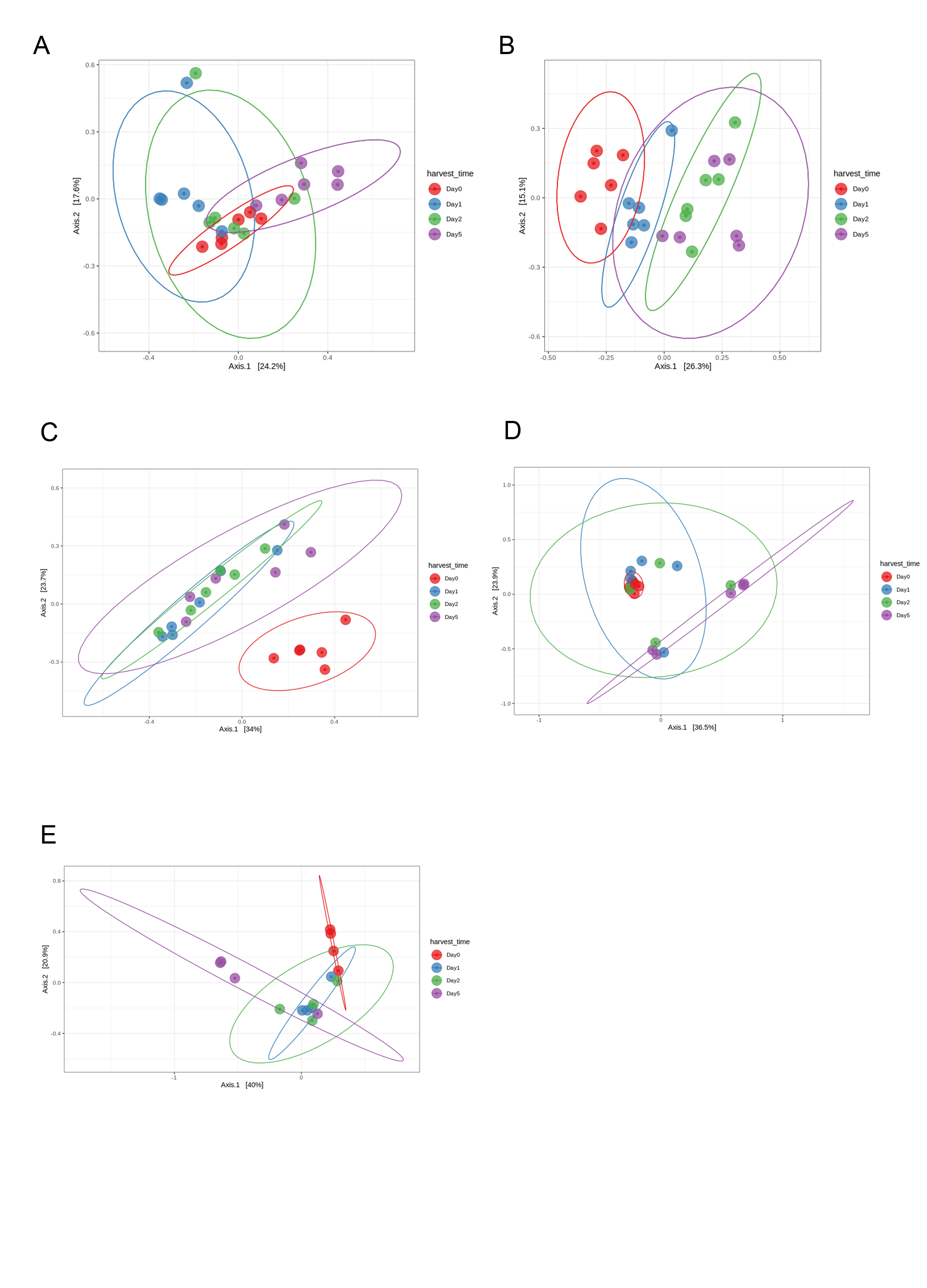

Supplement: Supplementary file 3 [file Image_2.TIF]

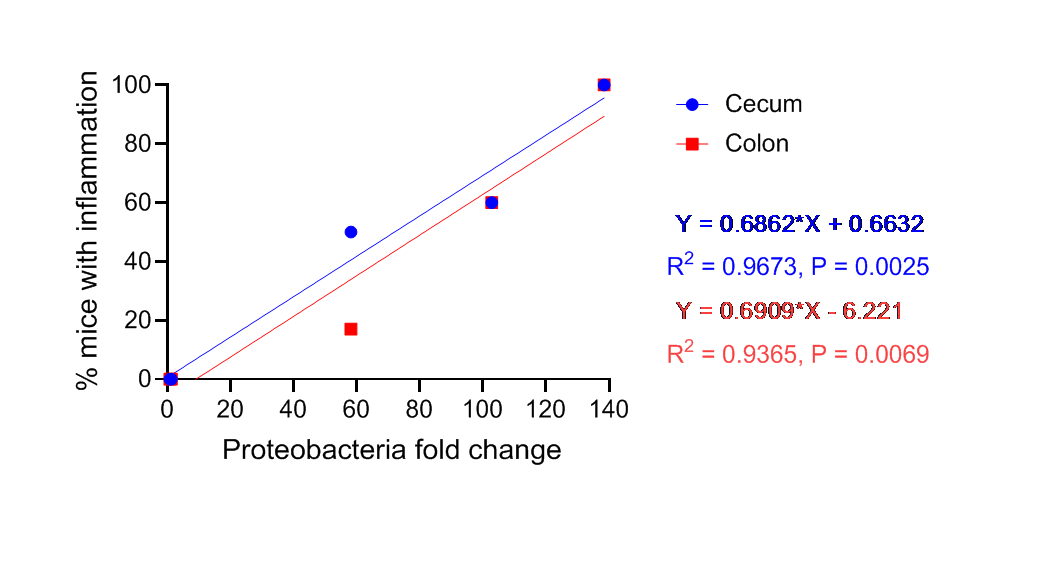

Supplement: Supplementary file 4 [file Image_3.TIF]
